# Performing a tarsorrhaphy

**Published:** 2015

**Authors:** Saul Rajak, Juliette Rajak, Dinesh Selva

**Affiliations:** Oculoplastic Fellow: South Australian Institute of Ophthalmology, Royal I Adelaide Hospital, Adelaide, Australia. Honorary lecturer: International Centre for Eye Health, London School of Hygiene and Tropical Medicine, London, UK.; Illustrator: Brighton, UK.; Professor of Ophthalmology: South Australian Institute of Ophthalmology, Royal Adelaide Hospital, Adelaide, Australia.

**Figure:**
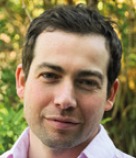
Saul Rajak

**Figure:**
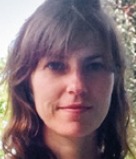
Juliette Rajak

**Figure:**
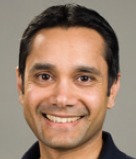
Dinesh Selva

## What is tarsorrhaphy?

Tarsorrhaphy is the joining of part or all of the upper and lower eyelids so as to partially or completely close the eye. Temporary tarsorrhaphies are used to help the cornea heal or to protect the cornea during a short period of exposure or disease. Permanent tarsorraphies are used to permanently protect the cornea from a long-term risk of damage. A permanent tarsorrhaphy usually only closes the lateral (outer) eyelids, so that the patient can still see through the central opening and the eye can still be examined.

## What are the indications for tarsorrhaphy?

To protect the cornea in the case of:

- inadequate eyelid closure, for example due to facial nerve palsy or cicatricial (scarring) damage to the eyelids caused by a chemical or burns injury
- an anaesthetic (neuropathic) cornea that is at risk of damage and infection
- marked protrusion of the eye (proptosis) causing a risk of corneal exposure
- poor or infrequent blinking, for example in patients in intensive care or with severe brain injuries.

To promote healing of the cornea in patients with:

- an infected corneal ulcer, which is taking a long time to heal
- non-healing epithelial abrasions.

Other indications include:

- To prevent conjunctival swelling (chemosis) and exposure after ocular surgery
- To retain a conformer or other device, for example in children with anophthalmia or adults after evisceration or enucleation.

## What are the different types of tarsorrhaphy?

The techniques for joining part or all of the upper and lower lids can be divided into short-term (temporary) and long-term (permanent) tarsorrhaphies. In both cases the procedure almost always involves using a suture to join the lids. Other techniques that are occasionally used are botulinum toxin tarsorrhaphy (the upper lid levator muscle is paralysed with the toxin), or the use of cyanoacrylate glue to join the lids and placing a weight (usually gold) in the upper lid.

**Figure 1a.**
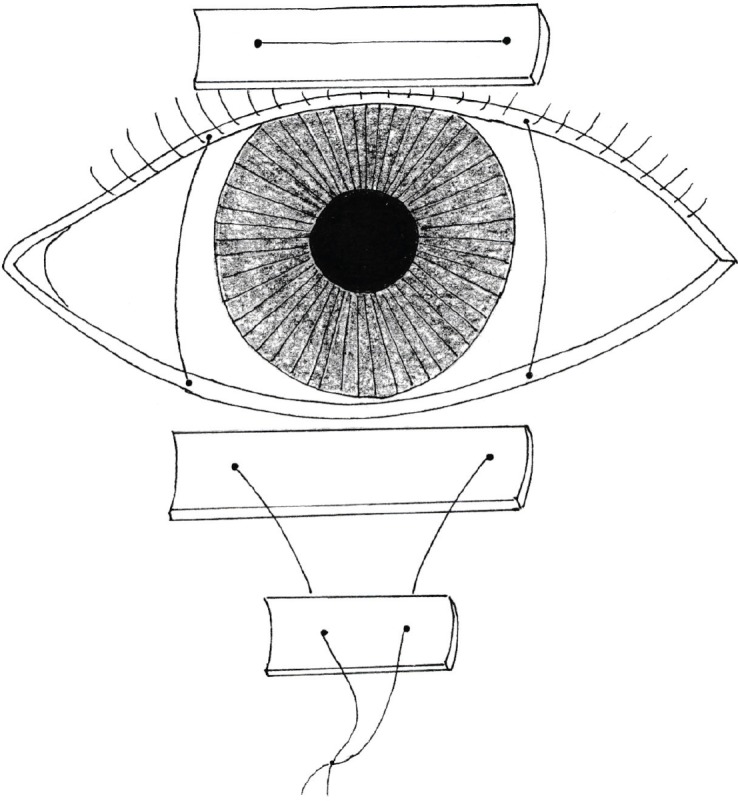
Alignment and threading of bolster

We will describe two simple procedures:

- A temporary central tarsorrhaphy with a drawstring that allows it to be repeatedly opened and closed for examining the eye.
- A permanent lateral tarsorrhaphy that leaves the central lids open, allowing the patient to see and the eye to be examined.

### The drawstring temporary central tarsorrhaphy (Figures 1a and 1b)

This simple suture tarsorrhaphy will be effective for 2–8 weeks.

1. Anaesthetise the central area of both the upper and lower eyelids with an injection of a few millilitres of local anaesthetic (e.g. lidocaine 1–2% or bupivacaine 0.5%). If anaesthetic with adrenaline is available it will reduce operative bleeding.
2. Clean the area with 5% povidone iodine. Leave the iodine for a few minutes. During this time prepare two x 2cm bolsters and one x 1cm bolster. The sutures are tied over the bolster (e.g. plastic tubing or small cotton wool balls) to prevent them cutting into the skin. They can be made from paediatric butterfly cannulas or other similar sterile plastic tubing. Cut each bit of tubing lengthwise to prepare a bolster ‘gutter’.
3. Pass a double-armed non-absorbable suture (e.g. silk, prolene or nylon 4-0, 5-0 or 6-0) straight through one of the 2cm bolsters, 2 mm from the end.
4. Line up the bolster in the middle of the upper lid and pass the same needle into the upper eyelid skin 3–4 mm above the lid margin, through the tarsal plate and out of the grey line of the lid margin. The grey line is the slightly darker line in the middle of the lid margin that is between the anterior and posterior lamellae of the lid.
5. Pass the same needle into the grey line of the lower lid, into the tarsal plate and out of the skin 2–3 mm below the lower eyelid margin.
6. Align the lower lid bolster centrally, and pass the needle through it a few millimetres from one end.
7. Pass the other needle of the suture through the upper bolster – upper lid – lower lid – lower bolster in the same way as the first needle, 2mm from the other end of each of the bolsters.
8. Pass both needles through the shorter length of bolster, 2mm from each end of the bolster (Figure 1a).

**Figure 1b.**
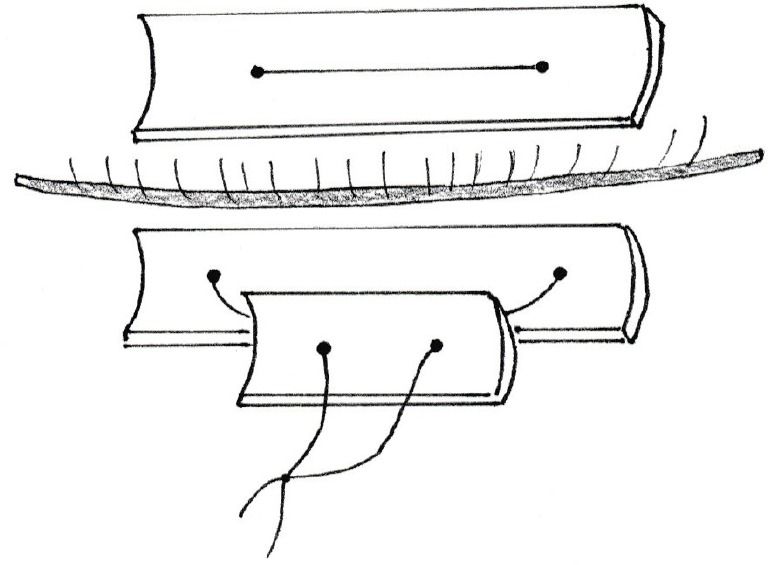
Using sutures and bolsters to close the eye
9. Slide the two lower lid bolsters upwards to close the eye. The smaller bolster ‘locks’ the lid closed (Figure 1b).
10. To separate the lids, pull the smaller bolster down and the lids will easily open.

If a single armed suture is being used, the needle can be passed from the lower bolster back up to the upper bolster.

### The permanent tarsorrhaphy (Figure 2a–f)

The upper and lower lids will not stay ‘stuck’ together when the sutures of a temporary tarsorrhaphy lose their tension after a few weeks. In a permanent tarsorrhaphy, some of the lid margin is debrided which allows the lids to stick together as they heal. Permanent tarsorrhaphies are almost always only lateral so that the patient can still see out of the central eyelid opening and the eye can still be examined. They should last at least 3 months (and sometimes forever).

The steps of a permanent lateral tarsorrhaphy are:

1. **Anaesthetise** the upper and lower lids as above.
2. **Split the anterior and posterior lamellae** (Figure 2a). Use a number 11 blade if available (or otherwise a number 15 blade) to cut along the grey line of the lateral third of the upper and lower lids to a depth of 2 mm. This will separate the anterior and posterior lamella. Continue the split inferiorly (lower lid) or superiorly (upper lid) for about 5 mm using either a blade or spring scissors. Make sure you keep the split parallel to the tarsal plate so that the eyelid neatly separates into anterior and posterior lamellae. The eyelid is likely to bleed and this can be controlled with a few minutes of pressure. Cautery can be used if available.
  **Figure 2a.**
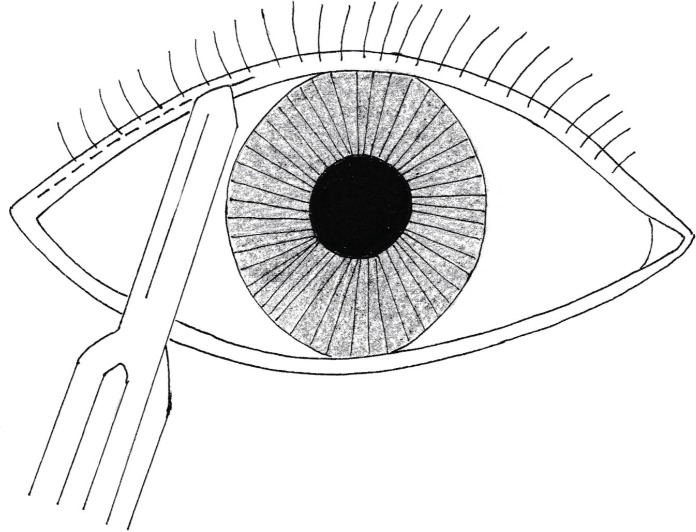
Splitting the anterior and posterior lamellae
3. **Excise 1 mm of the posterior lamella** (Figure 2b). This removes the epithelium of the lid margin and will enable the lids to stick together when they heal.
4. **Close the posterior lamella** (Figures 2c and 2d). Pass the needle of an absorbable 5-0 or 6-0 suture into the posterior lamella of the upper lid and then bring it out a little bit further along the upper lid posterior lamella. Pass the needle into the posterior lamella of the lower lid in line with the point of emergence on the upper lid. Pass the needle so that it emerges from the posterior lamella of the lower lid in line with where the needle was first inserted into the upper lid. Repeat this with a second suture.
5. **Close the anterior lamella (eyelid skin)** (Figure 2e). Insert a needle drawing a 4-0 to 6-0 sized thread into the skin of the upper lid, 2–3 mm above the lid margin and bring it out of the anterior lamella of the upper lid margin. Pass the needle directly across into the anterior lamella of the lower lid margin and out of the skin 2–3 mm below the lid margin. Tie the suture. Repeat this with several sutures placed 3 mm apart until the skin is closed over the closed posterior lamella.

When you have finished the procedure note the following two things (Figure 2f):

- If you have neatly joined the lateral third of the upper and lower eyelids, there will still bean opening that the patient can see through. The opening will obviously be narrower horizontally, but it will also be narrower vertically, which will give more protection to the cornea in the open area.
- In this procedure, the anterior lamella and eyelashes are undamaged – therefore if the tarsorrhaphy is opened at a later date, the lid will look almost normal. These tarsorrhaphies often last forever, but if they need to be divided this can be done by injecting some local anaesthetic and cutting the sutures.

**Figure 2b.**
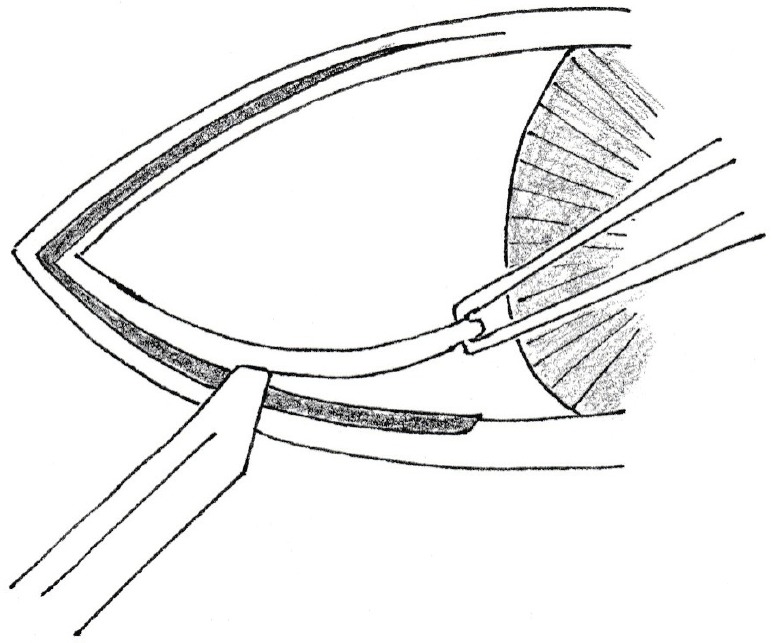
Excising 1 mm of the posterior lamella

**Figure 2c.**
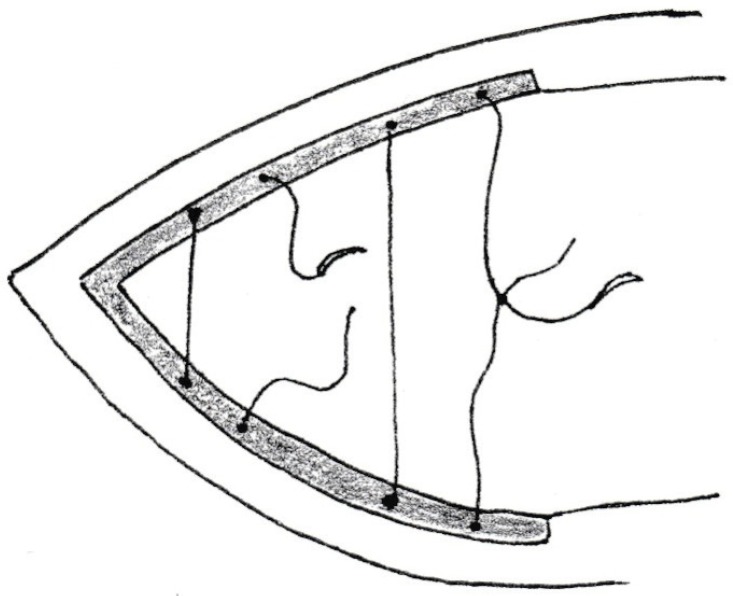
Closing the posterior lamella

**Figure 2d.**
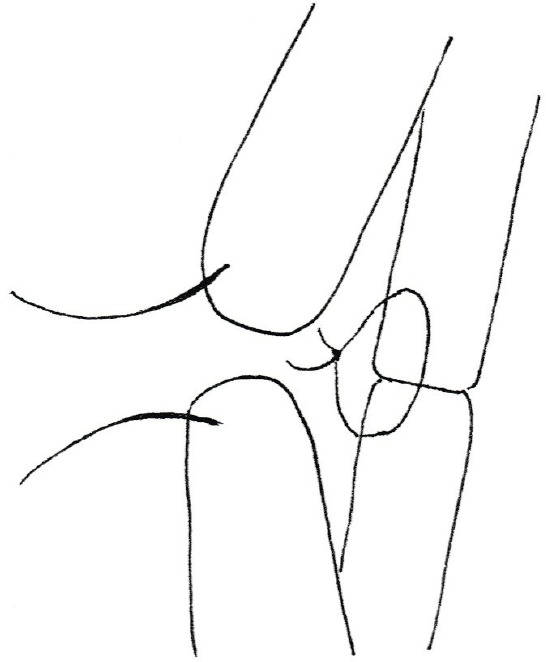
Closing the posterior lamella (side view

**Figure 2e.**
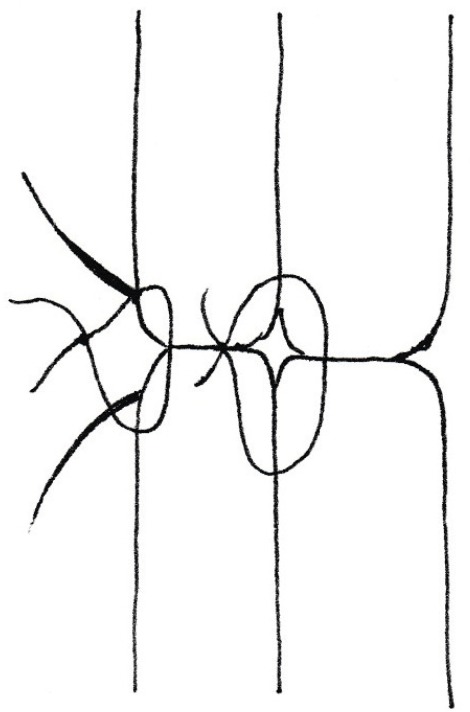
Closing the anterior lamella

**Figure 2f.**
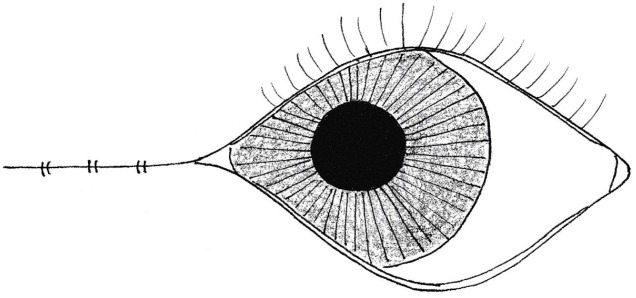
After the procedure

